# Homeostatic Plasticity of Subcellular Neuronal Structures: From Inputs to Outputs

**DOI:** 10.1016/j.tins.2016.08.004

**Published:** 2016-10

**Authors:** Winnie Wefelmeyer, Christopher J. Puhl, Juan Burrone

**Affiliations:** 1 Centre for Developmental Neurobiology, King's College London, New Hunt's House, Guy's Hospital Campus, London, SE1 1UL, UK

**Keywords:** homeostatic plasticity, axon initial segment, structural plasticity, dendritic spines, presynaptic terminals

## Abstract

Neurons in the brain are highly plastic, allowing an organism to learn and adapt to its environment. However, this ongoing plasticity is also inherently unstable, potentially leading to aberrant levels of circuit activity. Homeostatic forms of plasticity are thought to provide a means of controlling neuronal activity by avoiding extremes and allowing network stability. Recent work has shown that many of these homeostatic modifications change the structure of subcellular neuronal compartments, ranging from changes to synaptic inputs at both excitatory and inhibitory compartments to modulation of neuronal output through changes at the axon initial segment (AIS) and presynaptic terminals. Here we review these different forms of structural plasticity in neurons and the effects they may have on network function.

## Trends

Structural plasticity in response to chronic changes in activity occurs at synaptic inputs at the level of both excitatory and inhibitory compartments. Although opposite in direction, they act to normalise the overall activity of the network in a homeostatic manner.

The output of neurons is also influenced by important structural modifications. The axon initial segment, where action potentials are initiated, undergoes changes in either length or position to fine-tune neuronal excitability.

Presynaptic terminals, which provide the final stage in neuronal output through the release of neurotransmitter, also show structural alterations that match their postsynaptic partners.

Overall, there appear to be multiple forms of plasticity that occur during chronic changes in activity, which together serve to stabilise network function.

## Balancing Change and Stability

**Figure 1:**
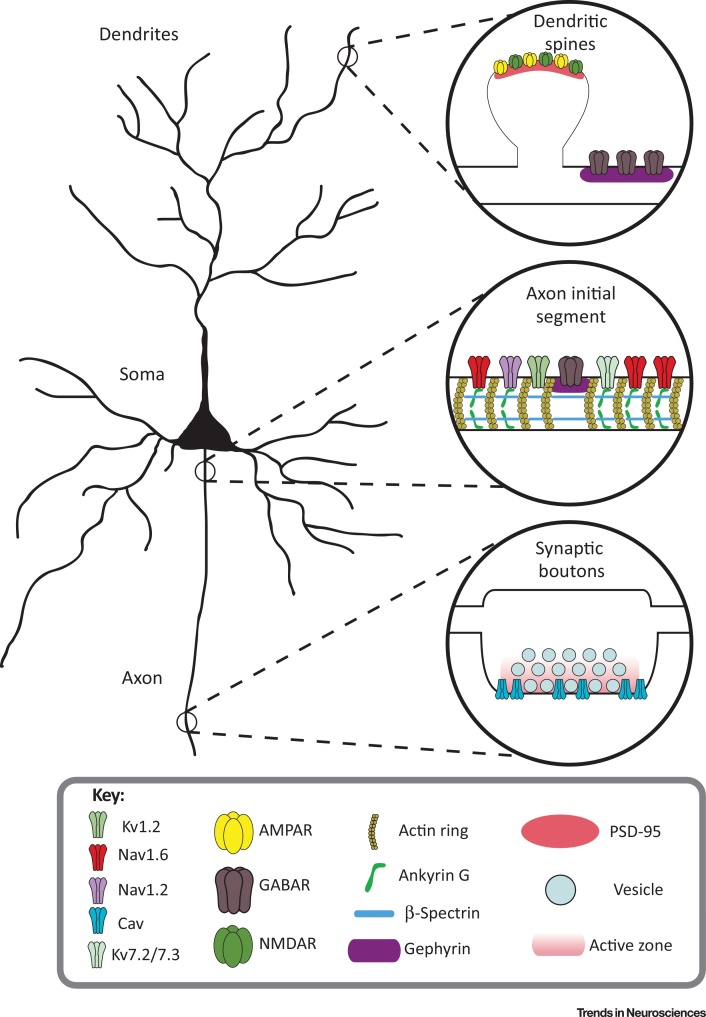
Structural Homeostatic Plasticity Occurs at Three Main Loci in Neurons. Inputs arrive primarily at the dendrites of neurons (top right), where synapses are formed on dendritic spines and the dendritic shaft. AMPA and NMDA receptors are located mainly in spines while inhibitory GABA_A_ receptors are found mostly on the dendritic shaft. At the axon initial segment (AIS) (middle right), ankyrin G tethers a large complement of voltage-gated channels, including Na_V_1.2, Na_V_1.6, K_v_7.2, and K_v_7.3, to the membrane. Inhibitory synapses are found localised to gephyrin in gaps between ankyrin G that also contain K_V_1.2. These components at the AIS initiate and shape neuronal output, which is transmitted along the axon. At presynaptic boutons (bottom right), activation of voltage-gated calcium channels by the action potential leads to exocytosis of neurotransmitter-filled vesicles at the active zone and thus transmission of the neuron's output signal.

Neurons come in all shapes and sizes [1]. A bird's-eye view of a neuron will show complex dendritic and axonal morphologies that vary dramatically depending on neuron type and developmental stage 2, 3. While these differing morphologies may underlie important functional roles for neurons in a given circuit, neurons also possess an immense capacity to change both their structure and function [4]. It is this capacity for change that allows neurons – and wider neural circuits – to store information and adapt to the environment. How neurons in the brain perform this job is an area of intense research that has yet to find a definite structural correlate. Whereas wholesale structural rearrangement of dendrites and axons occurs mainly during development, subtler changes occur in adult neurons, with the most compelling evidence so far suggesting that the number and strength of synaptic connections change in response to experience-driven neuronal activity 5, 6. These changes in synaptic connectivity are thought to be the principal form of information storage in the brain, endowing organisms with the ability to learn 6, 7. However, this high level of ongoing plasticity comes at a price. Highly plastic systems are inherently unstable, as positive feedback loops emerge that can drive networks to extreme levels of activity that are detrimental to the organism [8]. Neurons, therefore, have the complex task of finding the right balance between plasticity and stability. To solve the stability problem, different strategies exist that allow neurons to maintain their excitability within reasonable bounds – a set of safeguards designed to avoid extremes while retaining the ability to build a circuit and store information. Under the general term of homeostatic plasticity they encompass numerous subcellular modifications that ultimately control function at both the single-cell and network level. These structural modifications span a very broad spatial domain that can range anywhere from the subnanometre to the micrometre scale [9] and that require different imaging modalities. For example, structural changes that result in complete removal or addition of spines are easily visualised by light microscopy whereas changes in synaptic strength might be accompanied by subtler structural modifications at the molecular level that can be observed only with ultrastructural imaging. Here we will focus on structural alterations that lie beyond the molecular level and cause visible changes in neuronal morphology, since we currently have a more complete picture of their role in homeostatic plasticity both *in vitro* and *in vivo*. We review how both input and output structures in neurons are modulated to maintain relatively stable levels of neuronal excitability. Specifically, we focus on synaptic connections (inputs) and the AIS (output), both of which have been shown to undergo important structural modifications in response to chronic activity changes (Figure 1). Finally, we discuss the link between the structure and function of these subcellular compartments and their role in controlling the overall excitability of neurons and circuits.

## The Synapse

The chemical synapse is a bicellular unit comprising a presynaptic terminal and a postsynaptic compartment separated by a synaptic cleft. Although the cleft is only 20–30 nm across, the experimental gulf between the two sides of the synapse has historically been quite large. Mainly due to technical limitations, studies looking at structural forms of plasticity tend to focus on either postsynaptic or presynaptic elements but rarely the two together. The fact that there is a strong correlation between the structure 2, 3, 10, 11 and function of pre- and postsynaptic elements 4, 12, 13, 14 lends further credence to studies that focus on one compartment only, but care should be taken as this functional correlation may not hold true in all conditions 5, 6, 12, 15. We have therefore divided this section into its constituent parts: excitatory postsynaptic spines, inhibitory postsynaptic compartments and presynaptic boutons. From these studies a consensus view of the structural changes that occur at the synapse as a whole is gradually beginning to emerge.

### Postsynaptic Excitatory Compartments: Dendritic Spines

Dendritic spines are small protrusions that cover the dendrites of most vertebrate excitatory cell types. They typically comprise a neck that extends no more than 2 μm from the dendritic shaft and ends in a small bulb. Spines are thought to increase neuronal connectivity by allowing the dendrite to reach a larger number of axons within the same space 4, 6, 7.

From a functional perspective, dendritic spines allow cells to isolate their inputs and perform linear and nonlinear summation of inputs 8, 16. As the mediators of most of the excitatory inputs to neurons, spines possess a huge complement of postsynaptic receptors and signalling machinery. They exhibit variable sizes and shapes depending on their location on the dendritic tree 9, 17 and are also highly dynamic (Box 1). Because of their role as mediators of synaptic transmission, dendritic spines represent a possible target for controlling the inputs to a neuron and therefore the overall excitability of the cell.

With the development of *in vivo* imaging techniques, changes to dendritic spine turnover and density in response to long-term activity perturbation have been observed. For example, input deprivation in the barrel cortex via trimming every other whisker, also known as ‘chessboard deprivation’, was found to lead to increased turnover of dendritic spines of layer 5 (L5) pyramidal neurons without any change in the density of spines [18]. A similar study in the visual cortex found that monocular deprivation resulted in a doubling of the rate of spine formation and therefore an overall increase in spine density in L5 apical dendrites [19]. Together, these studies suggest that neurons employ different structural modifications at the level of excitatory inputs to adapt to their new condition, since both correlate with a recovery of neuronal function that can be seen as homeostatic in nature. Whereas sensory deprivation in the barrel cortex resulted in increased sampling of neighbouring whiskers [18], in the visual cortex it results in a biased increase in responsiveness to the spared eye 20, 21. In both cases these functional changes appear to coincide with the structural remodelling, suggesting that structural changes at the level of dendritic spines are involved in homeostatic upregulation of activity levels in the neuronal network. So far, however, a causative link between changes in structure and function is missing. Technically challenging experiments that simultaneously assess spine turnover across many days and overall levels of neuronal activity in the same cell will help provide a better link between the two. However, it is only by performing interventions (probably at the molecular level) that block any changes in spine dynamics following sensory manipulation that we will finally establish a causal bridge between structural plasticity and function.

The original descriptions of synaptic homeostatic plasticity suggested that all synaptic inputs to a neuron changed in a multiplicative manner, a concept known as synaptic scaling [22]. Given that spine size has been shown to correlate with the number of AMPA receptors and the strength of the synapse 23, 24, 25, synaptic scaling should be accompanied by structural changes of dendritic spines. This classic mechanism of firing rate homeostasis has recently been observed structurally. Keck *et al.* [26] conducted long-term imaging of spines after bilateral retinal lesions. They observed a recovery of activity after lesions mirrored by an overall increase in spine size. Interestingly, these structural changes were multiplicative in nature. In this way, any information encoded in the heterogeneity of synaptic strengths will be preserved while at the same time renormalising the firing rate of the neuron. It is important to note that this cell-wide scaling effect has not been seen in all preparations or experimental paradigms and alternative, more local forms of plasticity have been proposed 9, 27 that have some experimental backing 28, 29. An interesting observation from the retinal lesion experiments was that the initial decrease in neuronal activity was only about 50% of non-deprived cortex, showing that homeostatic plasticity can be invoked *in vivo* without the need to completely silence networks, as is generally the case in *in vitro* studies.

Although the above studies were conducted with excitatory neurons, changes in dendritic spines are also observed in a small subpopulation of interneurons that carry dendritic spines [30]. After focal retinal lesions, spines in the lesion projection zone were monitored in L1 and L2/3. Spine turnover increased during the first 72 h and ultimately led to a decrease in spine density due to a lower survival rate for these spines. This finding is interesting from both a mechanistic and a network point of view. Interneurons appear to respond very differently to chronic changes in activity compared with neighbouring excitatory pyramidal cells by reducing instead of increasing their excitatory synaptic drive, suggesting clear differences in the activity-dependent mechanisms of spine plasticity. Functionally, this difference in response makes sense for the network as a whole since it will simultaneously increase excitation to principal neurons while dampening excitation in inhibitory neurons, allowing the network to recover from its slump in activity [30]. An interesting point to note here is the time course of spine remodelling after sensory deprivation. Compared with spiny interneurons, excitatory cells generally exhibit structural plasticity with a longer delay, not within hours but rather within days, and lasting for weeks to months 18, 19, 31, 32. This suggests that structural plasticity of interneurons may be the first step in reorganising the cortical network after sensory deprivation. Finally, although dendritic spines are clearly a hotspot for structural plasticity, excitatory inputs that arrive directly on the dendritic shaft, as is the case for most spineless interneurons, may also undergo substantial rearrangements. However, partly because of the lack of a clearly visible structure, much less is known about them.

### Inhibitory Postsynaptic Compartments

**Figure 2:**
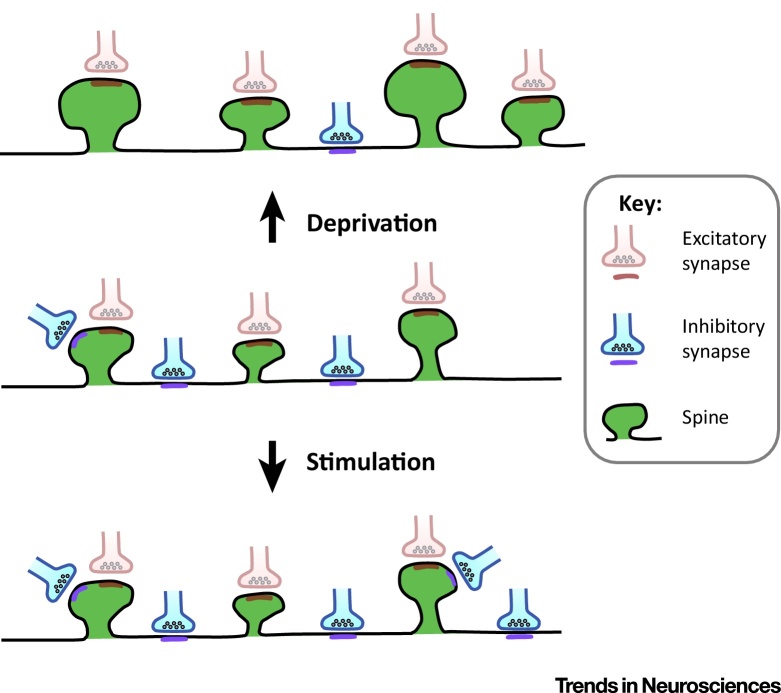
Inhibitory Synapses Formed on Pyramidal Cell Dendrites Show Homeostatic Plasticity in Response to Modulation of Activity Levels. At rest (middle), inhibitory boutons synapse mainly onto the dendritic shaft, although some innervate dendritic spines [34]. After sensory deprivation (top), inhibitory synapses are removed from both the dendritic shaft and the spines 34, 35, 37. At the same time, spine size and density increase 19, 26. Conversely, after long-term sensory stimulation, the density of inhibitory innervation on both shafts and spines increases [40] (bottom).

The study of inhibitory synapses suffers from a similar problem to that of excitatory shaft synapses, which is that there is no obvious structural correlate for them. To overcome this problem, overexpression of fluorescently tagged gephyrin, the scaffolding protein found at inhibitory synapses, has now become the method of choice to label inhibitory postsynaptic compartments, although other labelling techniques have recently been developed [33]. Using this approach, Chen *et al.* [34] and van Versendaal *et al.* [35] were able to follow inhibitory synapse dynamics *in vivo*. Interestingly, almost a third of all dendritic inhibitory synapses onto L2/3 pyramidal neurons were found to form directly on dendritic spines rather than on the dendritic shaft [34] (Figure 2). Overall, less than 3% of spines in L2/3 are thought to receive inhibitory inputs [36], which were found to be distributed in a biased manner along the dendritic tree with almost twice as many found in distal apical dendrites compared with proximal locations [34]. These spines, dually innervated by inhibitory and excitatory synapses, were found to be highly persistent both before and during monocular deprivation and may even receive specific inputs that arrive from thalamic neurons [36]. Although inhibitory structures in general were found to be highly dynamic, inhibitory spine synapses showed a larger turnover rate during monocular deprivation compared with their shaft equivalents 34, 35, 37 although the spine itself remained stable. The physiological effect of inhibitory spine synapses is thought to be highly compartmentalised [38], potentially allowing an inhibitory input to veto the local excitatory synaptic signal. The rapid removal of inhibitory spine synapses may thus result in disinhibition of the subcortical excitatory drive that arrives on these spines and be part of the mechanism that strengthens responsiveness to the non-deprived eye. Loss of shaft synapses, by contrast, will have a more general effect on neuronal activity levels, influencing dendritic integration more globally along a dendritic branch [16]. The removal of inhibitory synapses argues for homeostatic control of neuronal activity that can act either locally or more globally depending on the subcellular location of the inhibitory contact and selectively influence excitatory drive on pyramidal neurons (Figure 2). The balance between excitation and inhibition is thought to be tightly controlled, at least in part, by the activity-dependent remodelling of inhibitory inputs [39], regulating the overall levels of neuronal excitability. In line with this, increases in sensory input through persistent single-whisker stimulation showed a calcium-dependent increase in inhibitory synapse numbers in the barrel cortex – specifically, those formed on spines – in agreement with a bidirectional role of activity in shaping inhibitory inputs to stabilise neuronal activity 40, 41.

### Presynaptic Boutons

Much of the focus in this review as well as in the literature has been on postsynaptic rearrangements. However, several elegant *in vivo* experiments have shown that axons and presynaptic boutons also have the capacity for change 30, 42, 43, 44, 45, 46, 47, 48 and often go hand in hand with postsynaptic plasticity.

Clear examples of parallel pre- and postsynaptic alterations were observed in the visual cortex. Following retinal lesions, the reduction of excitatory dendritic spines on GABAergic spiny interneurons was accompanied by a decrease in the number of boutons along their axons and a corresponding loss of inhibitory input to L5 pyramidal cells [30]. Similarly, following eyelid suture, interneurons in L2/3 retracted a number of their dendritic branch tips, which was accompanied by elimination of interneuron boutons leading to loss of inhibitory input to neighbouring pyramidal cells [46]. This suggests that the structural plasticity of dendritic inputs and axonal outputs in interneurons is part of a homeostatic mechanism that serves to increase activity levels in subnetworks of neurons located in the lesion projection zone (LPZ). Inhibitory interneuron boutons were also found to disappear in the barrel cortex in response to whisker plucking [45]. More dramatically, interneurons located in the deprived barrel column underwent large-scale axonal remodelling, extending axon collaterals outside their column into the non-deprived, neighbouring cortical area. This structural remodelling was able to more than double the length of the typically short interneuron axons. Marik *et al.* [45] also examined the axonal projections of L2/3 excitatory pyramidal neurons, which also exhibited extensive reorganisation. Pyramidal neurons located outside the deprived barrel column extended their axons into the deprived cortical area and started sprouting, strongly increasing the local axonal density after 2 weeks of whisker plucking. This was accompanied by an increase in excitatory bouton density, which contrasted with the decrease observed in interneurons. A similar pattern of axon growth was also found in the monkey primary visual cortex after focal binocular retinal lesions, where axons of pyramidal cells extended into the LPZ [44] and inhibitory neurons located inside the LPZ elongated their axons to project to the peri-LPZ [48]. It remains to be shown whether the interneurons projecting out of the LPZ form synaptic connections with those pyramidal cells that now project into the LPZ. Nevertheless, these concurrent changes at the axons of excitatory and inhibitory cells suggest that there is a homeostatic drive to maintain an appropriate balance between the amount of excitation and inhibition (the E/I balance) in the network.

Presynaptic structural changes can also serve to homeostatically regulate the E/I balance at the single-cell level [49]. After motor learning, new spines are formed on the distal apical dendrites of pyramidal cells. This is accompanied by local loss of inhibitory boutons formed by somatostatin-positive interneurons, which preferentially contact the distal apical dendrite. At the same time, parvalbumin-positive interneurons increase the number of boutons and thus the synapses that contact the soma and proximal dendrites of pyramidal cells. These complementary structural changes of different interneuron types allow the pyramidal cell to form new spines thanks to the local disinhibition, without compromising the E/I balance and thus risking hyperexcitability.

Overall, it is now becoming clear that presynaptic structural changes, at the level of the bouton as well as the axonal arbour, can serve to homeostatically regulate excitability levels after sensory deprivation as well as learning. This goes hand in hand with homeostatic alterations of neurotransmitter release probability 11, 50, 51. However, we still know comparatively little about presynaptic modifications and how these complement postsynaptic structural plasticity. In particular, the relatively small number of studies on presynaptic homeostatic plasticity leaves us speculating how modifications might differ between different cell types. As discussed above, we are slowly gaining insight into how changes in inhibitory and excitatory neurons complement each other. However, there are many different cell types within the broad categories of inhibitory and excitatory neurons, with vastly differing functional roles in the network. Interestingly, baseline bouton dynamics can vary strongly between excitatory cell types, with thalamocortical axonal boutons proving remarkably stable compared with intracortical axonal boutons [42]. It would be particularly interesting to see how these bouton dynamics change in response to a homeostatic challenge. As we gather more information on presynaptic structural plasticity we will gain a better understanding of the different structural modifications across different cell types and their interactions for network-level homeostasis.

## The AIS

**Figure 3:**
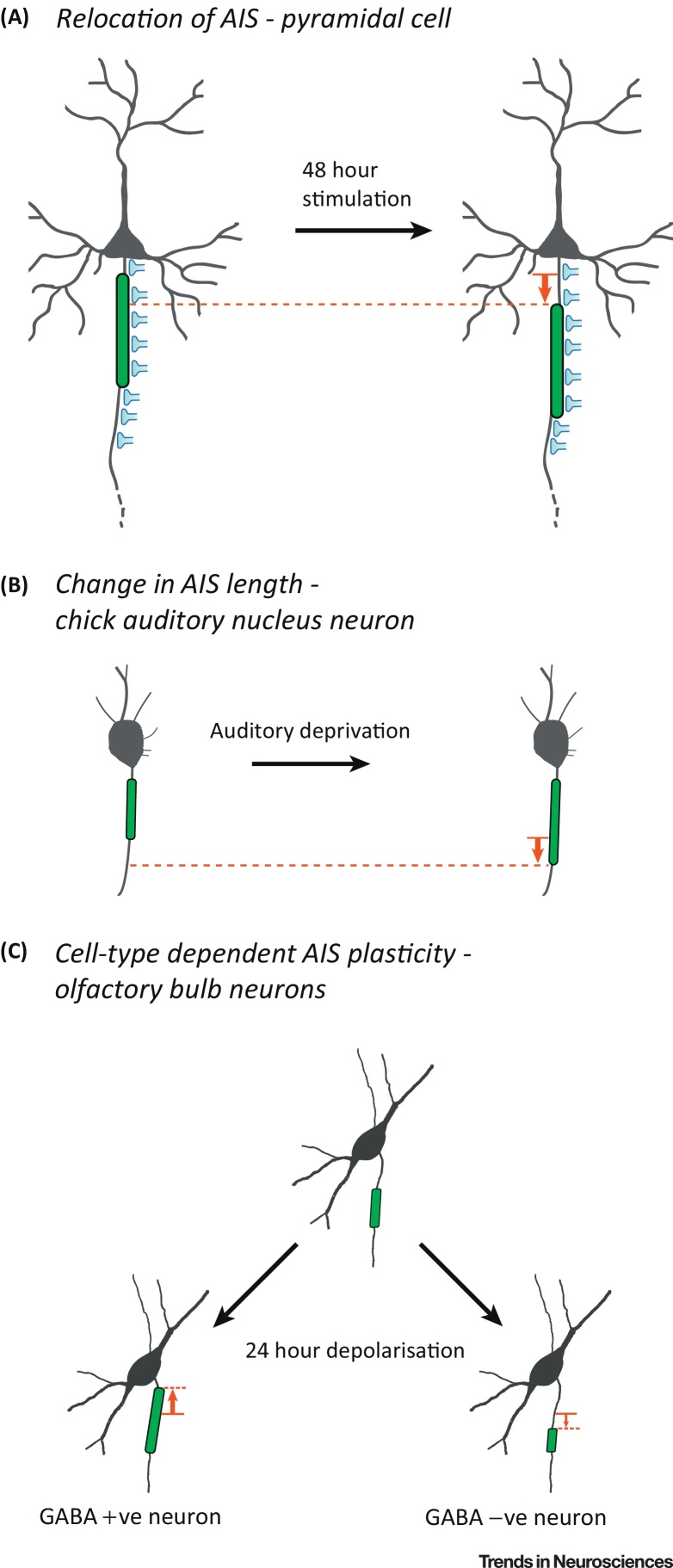
Cell Type-Specific Structural Plasticity of the Axon Initial Segment (AIS). (A) Pyramidal cells move their AIS away from the soma after long-term stimulation 52, 57, 58, 59. Importantly, GABAergic axoaxonic inputs to the AIS do not change, creating a partial mismatch between the two compartments and an overall decrease in excitability [58]. (B) Auditory deprivation increases the length of the AIS in chick auditory nucleus neurons resulting in an increase in excitability [53]. (C) Following 24 h of depolarisation, GABAergic olfactory bulb interneurons show proximal lengthening of their AIS, which brings it closer to the soma. By contrast, neighbouring non-GABAergic interneurons respond by shortening their AIS instead, resulting in a more-distal start position [63].

Structural plasticity at the axon is not restricted solely to boutons. Two studies in 2010 demonstrated that structural plasticity can occur at the heart of the site responsible for action potential generation: the AIS 52, 53. The AIS is located at the proximal end of the axon, close to the soma, and is densely populated by various proteins including voltage-gated sodium and potassium channels. Due to the high density of these cation channels 54, 55 and the local properties of the axon [55], the AIS is the location in the cell with the lowest threshold for action potential generation [56] and is responsible for integrating synaptic inputs to elicit a spike. It is perhaps unsurprising, therefore, that changes in the structure of the AIS can have important consequences for neuronal excitability in general, making it a prime site for modulating neuronal output. A change in length as well as a change in position along the axon were originally reported in response to chronic alterations in neuronal activity and have been proposed to act as stabilisers of neuronal excitability 52, 53 (Figure 3). For example, auditory deprivation achieved by removal of the cochlea in chicks resulted in a large drop in excitatory drive to the auditory nucleus magnocellularis. Crucially this also led to elongation of the AIS by more than 50% in neurons within this nucleus. This increase in AIS length reflected an increase in the number of voltage-gated sodium channels and an overall increase in neuronal excitability [53]. In hippocampal neurons, a different strategy is used. It is now well established that either 2 days of optogenetic photostimulation or high potassium depolarisation leads to a cell-autonomous outward shift of the AIS in excitatory neurons 52, 57, 58, 59, accompanied by a reduction in intrinsic excitability 52, 59. This form of structural plasticity that spatially translocates the AIS to a more distal domain in response to increased activity is dependent on calcium influx through L-type calcium channels and activation of the calcium-sensitive phosphatase calcineurin [57]. Regardless of the mechanism employed, both of these structural forms of AIS plasticity appear to serve a homeostatic purpose, bringing the cell's excitation levels closer to the state before the manipulation. It is important to note, however, that the effect of changes in AIS position on excitability are likely to depend on the cell type or model used [60]. A study of L5 cortical neurons showed that AP initiation occurred in the distal portion of the AIS, as this was sufficiently distant from the large conductive and capacitive load of the soma and dendrites [55]. As a result, distal relocation of the AIS away from the large L5 pyramidal cell somatodendritic domain and towards the thinner, myelinated part of the axon would be predicted to result in an increase in excitability 55, 61. However, myelination is strongly variable [62] and many cell types, including hippocampal pyramidal cells in culture, have largely unmyelinated axons and much smaller cell bodies. This could possibly explain why in some cases the dissipation of current along the axonal membrane outweighs the uncoupling from the somatodendritic current sink [60], such that a distal shift of the AIS can decrease excitability instead [52]. In any case, this strongly argues for an important contribution of structural parameters such as axon diameter, soma size, and, of course, AIS length and location to neuronal output generation.

One intriguing finding in hippocampal neurons is that interneurons do not show any activity-dependent changes in AIS structure [52]. There are a several reasons why this might be the case, including the possibility that interneurons may require different levels or patterns of activity compared with excitatory neurons or simply the fact that the diversity of interneuron types may occlude AIS plasticity if not studied in a specific neuronal class. More recently, homeostatic AIS plasticity has been observed in a subset of dopaminergic interneurons in the olfactory bulb [63] (Figure 3C). Following 24 hours of depolarisation of cultured olfactory bulb neurons, the AIS lengthened and moved towards the soma. This is opposite to the effect seen in neighbouring excitatory olfactory bulb neurons, where the AIS moved distally and became shorter, and also to the distal AIS relocation observed in excitatory cells in the hippocampus. This reverse structural plasticity of the AIS in interneurons might therefore act in concert with the activity-induced AIS changes observed in excitatory cells to homeostatically regulate activity within the neuronal network. However, Chand *et al.* [63] observed only a trend towards decreased excitability in these dopaminergic interneurons, which complicates the functional significance of this structural modification. Nevertheless, together these results once again show how similar perturbations in the activity of a neuron or network can lead to opposite structural forms of plasticity in excitatory versus inhibitory interneurons.

### The AIS and its Synapses

Interneurons can also play a role in modulating action potential generation at the AIS of pyramidal cells. In particular, a specific type of parvalbumin-positive, fast-spiking interneuron, the Chandelier cell, is responsible for forming rows of GABAergic synapses along the AIS in the cortex and hippocampus [64]. A single chandelier cell has a vast axonal arbour that can form synapses with the AIS of many hundreds of neighbouring pyramidal neurons and is therefore likely to play an important role in modulating network function. However, our knowledge of these interneurons is remarkably limited, mainly due to the fact that they are only sparsely found in the brain and, until recently, could not be labelled specifically by transgenic mouse lines. As a result, the precise role they play in neuronal integration and excitability, as well as in behaviour and network function, remains unclear and, in some cases, controversial 65, 66 (for a review see [64]). Recent work has explored activity-dependent forms of plasticity at the AIS to establish whether these axoaxonic synapses follow the structural modifications described above. Surprisingly, relocation of the AIS after chronic stimulation did not result in a change in synapse location in either dissociated hippocampal neurons or hippocampal slices 58, 59, resulting in a mismatch between the two. From a functional point of view, modelling results revealed that this arrangement actually helps to reduce neuronal excitability levels. Keeping axoaxonic synapses in their position despite an outwards shift of the AIS results in a greater number of GABAergic synapses in the region of the axon proximal to the soma, preceding the site of action potential generation. At the same time, synapses that were originally located beyond the AIS ensure that the number of axoaxonic contacts on the AIS remains constant. This leads to an increase in the inhibitory effect of axoaxonic synapses [59] and raises interesting questions regarding their arrangement at the nanoscale level (Box 2). In addition to the position of the synapses along the proximal axon, the exact effect that Chandelier cells exert on pyramidal cell excitability will also depend on their activity levels and the strength of the axoaxonic synapses. With the recent development of transgenic mouse lines that allow preferential labelling of Chandelier cells 65, 67, the role that these intriguing interneurons play in the control of network activity and the modulation of pyramidal cell output will surely grow in leaps and bounds.

## Linking Structure and Function

Neurons appear to have a large arsenal of diverse tools that they can employ to control their overall levels of excitability. In this review we have described several different forms of structural plasticity that will alter neuronal function in very distinct ways. What is less clear is how (and when) these forms of plasticity are employed by neurons to regulate circuit function and behaviour. Very few studies consider the interaction between the different forms of homeostatic plasticity [68]. For example, it is as yet unknown whether the structural plasticity of the AIS and the changes in excitatory and inhibitory synaptic boutons as well as spine dynamics cooperate to homeostatically adapt activity levels in single neurons. Unsurprisingly, an important question in the field is to try to merge these forms of plasticity to understand how neurons control their excitability. Do they occur simultaneously or do they follow each other in a temporal sequence where successive control mechanisms are implemented until excitability is brought under control? A temporal response sequence is reminiscent of the initial plasticity of inhibitory synapses followed by modifications of excitatory inputs observed in response to sensory deprivation 18, 19, 30, 31, 32. Measuring the time constants of different forms of homeostatic plasticity and their temporal relation to each other, as well as understanding the pathways that elicit each form of plasticity, will therefore be an important step in understanding how these mechanisms come together.

Even less is known when we consider homeostatic plasticity at the circuit level, since different cell types might use different complements of mechanisms depending on their functional role in the network. This is nicely exemplified by the different and often opposite adjustments that inhibitory and excitatory neurons make to rebalance network activity after a challenge such as sensory deprivation 30, 45, 63. Further, even within the broad categories of excitation and inhibition there are many different subtypes of neurons with distinct functional roles in the network 69, 70. For example, interneurons that provide feedforward inhibition onto pyramidal cells may respond differently to those that cause disinhibition by synapsing onto other interneurons. Here, cell identity may be the important factor that controls the direction and type of homeostatic plasticity employed, with the overall goal of controlling network activity as a whole. We are thus only at the start of understanding the full complement of compensatory mechanisms and their interactions.

Even when considering single, well-defined structural plasticity phenomena, we have much to learn regarding their influence on neuronal function. For example, although forming new spines and gaining new synaptic inputs is likely to increase overall neuronal activity levels, the exact functional consequences will depend on the morphology of the spines, the strength of the synapse, and the release probability of the presynaptic cell and its activity levels, as well as the location of the synapse along the dendrite [71]. Equally, relocation of the AIS is predicted to change excitability levels and has indeed been shown to correlate with a change in neuronal output 52, 59. However, whether this structural plasticity is sufficient to explain the functional change in neuronal output or whether other alterations are necessary, such as in ion channel composition, conductance, or distribution, is unclear. More work is needed to bridge the gap between structural plasticity and its consequences for neuronal function.

## Concluding Remarks

It is becoming increasingly clear that structural forms of plasticity can occur at both input and output compartments, during both learning and homeostatic adaptation. Interestingly, it has also become apparent that inhibitory and excitatory neurons undergo complementary forms of structural plasticity, maintaining an optimal E/I balance and ultimately regulating network excitability. Yet many questions remain unanswered (see Outstanding Questions). Do excitatory and inhibitory neurons use different sensors and effectors of neuronal or network activity to bring about complementary forms of structural plasticity? How do different forms of homeostatic plasticity, at either the synapse or the AIS, come together? Do they occur simultaneously or is there some hierarchy in the type of structural modification employed? Each will alter the input–output relation of the neuron or network in a different way, which raises the possibility that the type of stimulus or the level of network activity may bias towards specific forms of structural plasticity. It is by increasing our knowledge of these different changes in subcellular structures, as well as the mechanisms that drive them, that we will gradually shed light on the role they play in controlling the overall activity levels of local circuits in the brain.

Outstanding Questions How do the multiple forms of structural plasticity described in this review come together? Each will have very different effects on neuronal function and they could therefore be used under different conditions. Do they make use of similar molecular pathways or is each one recruited independently?

How do modifications of excitatory neurons differ from those of inhibitory neurons? Considering their often opposite responses to chronic changes in activity, do the sensors and effectors for homeostatic plasticity differ between the two cell types?

Following from this, do different types of interneurons show distinct forms of plasticity depending on whether they form connections with principal neurons or with other inhibitory neurons? From the point of view of network stability, disinhibitory loops may respond differently to feedforward inhibitory circuits.

More specifically, how local are the structural modifications of dendritic spines? Do they scale along the entire cell or is homeostatic plasticity confined to more local, dendritic domains? Do inhibitory neurons that do not possess dendritic spines show similar forms of plasticity?

Are the structural modifications of the AIS sufficient to explain the observed changes in neuronal excitability or do other events also play a role, such as plasticity of ion channel properties? How does the local inhibitory circuit of axoaxonic synapses at the AIS modulate neuronal output during AIS plasticity?

Finally, over what spatial scales does structural plasticity of neuronal compartments occur? Although our focus in this review was restricted to compartments that can be resolved by conventional microscopy techniques (larger than 1 μm), there could be subtler changes that occur at the nanoscale level. For example, changes in the structure of the active zone, the postsynaptic density, or the distribution of channels along the AIS could all lead to important changes in neuronal function that could be assessed by super-resolution imaging techniques.

## Box 1 Turnover of Dendritic Spines

Dendritic spines are not static; they grow, retract, shrink, and form anew throughout the lifetime of an organism [72]. These turnover rates vary between neuron types and can be affected by learning, disease, and sensory experience. The balance between formation and elimination of spines determines the density of spines along a dendritic branch. Thus, while the density of dendritic spines may be constant, turnover rates can be relatively high provided the rates of formation and elimination are balanced.

Following the development of *in vivo* imaging techniques, the morphology of spines has been tracked in the intact brain and even in awake, behaving mice. Two pioneering studies in this area 18, 73 repeatedly imaged dendritic spines in cortical neurons to provide the first measurements of turnover rates *in vivo*. The overall conclusion from these experiments was that cortical neurons appeared to have two populations of spines: those that remained stable and did not turn over and a more dynamic set of spines that showed rapid turnover rates of a few days. Interestingly, a more recent study using two-photon microendoscopy to image pyramidal cell dendritic spines in the hippocampus showed a distinct lack of stable spines, all of them turning over with a mean lifetime of 1–2 weeks [74]. In addition, the developmental stage of the organism also affects spine turnover 40, 75, 76, such that younger animals have a much larger proportion of dynamic spines compared with the more stable spines found in adults.

## Box 2 Nanoscale Structure of the AIS

Recent super-resolution imaging studies showed clusters of GABA_A_ receptors (GABA_A_Rs) and the voltage-gated potassium channel Kv2.1 along the AIS, present in pockets that did not contain any ankyrin G, the main organiser of the AIS [77]. This intriguing arrangement, where the AIS structure provides ‘holes’ for the synapses to occupy, suggests the coexistence of two independent molecular domains, one that provides the scaffolding for recruiting proteins to the AIS and another for axoaxonic synapses. Each of the two domains can even undergo structural forms of plasticity irrespective of the other, such that synapses remain in place when the AIS dislocates 58, 59 and raising the question of whether the holes also shift position relative to the AIS. Finally, the AIS, the axon, and sometimes even dendrites have been shown to be populated by a ring-like periodic lattice structure comprising actin, spectrin, and other associated molecules that presumably acts to improve the stability of these thin, long processes 78, 79, 80, 81. However, ankyrin G periodicity is not as perfect as that of actin or βIV-spectrin [80], which may reflect the synaptic holes described above. In light of the large-scale structural forms of plasticity at the AIS, it will be important to understand how this highly regular lattice structure is modified by neuronal activity and whether its disassembly or a subtler modification is required for AIS plasticity.
